# Increased Levels of Human Carotid Lesion Linoleic Acid Hydroperoxide in Symptomatic and Asymptomatic Patients Is Inversely Correlated with Serum HDL and Paraoxonase 1 Activity

**DOI:** 10.1155/2012/762560

**Published:** 2012-05-29

**Authors:** Elad Cohen, Michael Aviram, Soliman Khatib, Asaf Rabin, Dalit Mannheim, Ron Karmeli, Jacob Vaya

**Affiliations:** ^1^Oxidative Stress Research Laboratory, Migal-Galilee Technology Center and Tel Hai College, P.O. Box 831, Kiryat Shmona 11016, Israel; ^2^Rappaport Family Institute for Research in the Medical Sciences, Rambam Medical Center, Haifa 31096, Israel; ^3^Department of Vascular Surgery, Carmel Medical Center, Haifa, Israel

## Abstract

Human carotid plaque components interact directly with circulating blood elements and thus they might affect each other. We determined plaque paraoxonase1 (PON1) hydrolytic-catalytic activity and compared plaque and blood levels of lipids, HDL, PON1, and HbA1c, as well as plaque-oxidized lipids in symptomatic and asymptomatic patients. Human carotid plaques were obtained from symptomatic and asymptomatic patients undergoing routine endarterectomy, and the lesions were ground and extracted for PON activity and lipid content determinations. Plaque PONs preserved paraoxonase, arylesterase, and lactonase activities. The PON1-specific inhibitor 2-hydroxyquinoline almost completely inhibited paraoxonase and lactonase activities, while only moderately inhibiting arylesterase activity. Oxysterol and triglyceride levels in plaques from symptomatic and asymptomatic patients did not differ significantly, but plaques from symptomatic patients had significantly higher (135%) linoleic acid hydroperoxide (LA-13OOH) levels. Their serum PON1 activity, cholesterol and triglyceride levels did not differ significantly, but symptomatic patients had significantly lower (28%) serum HDL levels and higher (18%) HbA1c levels. Thus LA-13OOH, a major atherogenic plaque element, showed significant negative correlations with serum PON1 activity and HDL levels, and a positive correlation with the prodiabetic atherogenic HbA1c. Plaque PON1 retains its activity and may decrease plaque atherogenicity by reducing specific oxidized lipids (e.g., LA-13OOH). The inverse correlation between plaque LA-13OOH level and serum HDL level and PON1 activity suggests a role for serum HDL and PON1 in LA-13OOH accumulation.

## 1. Introduction

The human atherosclerotic plaque is characterized by increased levels of oxidized lipoproteins, such as LDL, HDL, phospholipids, triglycerides [1, 2], oxidized cholesterol products (oxysterols) [3], FFAs, and fatty acid derivatives [4], as well as proteins such as fibrinogen, apolipoprotein A-I (apoA-I), clusterin, and paraoxonase (PON) [5, 6]. Accumulating cholesterol in the plaque tends to precipitate and crystallize, forming sharp edges with increased volume leading to rupture-prone fibrous caps [7]. These “vulnerable plaques” have several features that differentiate them from their nonvulnerable counterparts: vulnerable plaques are less stable, characterized by a large lipid core, thin fibrous cap and less collagen, intraplaque hemorrhaging and infiltration of inflammatory cells without calcification [8]. These unstable plaques are associated with increased ischemic events, and about 20% of ischemic strokes are attributed to carotid artery atherosclerosis. Differentiating asymptomatic from symptomatic patients is important in determining the appropriate treatment. Major serum biomarkers of plaque vulnerability are related to inflammatory and proteolytic markers, yet no biomarkers exist for regular clinical use to indicate high risk of neurological events and to select patients for carotid surgery [8].

Mammalian PONs (PON1, PON2, and PON3) are a unique family of calcium-dependent esterases/lactonases. Many of the antiatherogenic properties of HDL are attributed to PON1. This enzyme reduces macrophages' cellular oxidative stress, decreases cholesterol-biosynthesis rate, and stimulates HDL-mediated macrophage cholesterol efflux, thus protecting against foam-cell formation and atherogenesis [9, 10]. PON1-deficient mice are susceptible to the development of atherosclerosis [11], whereas overexpression of human PON1 in mice inhibits atherosclerosis development [12]. Epidemiological evidence demonstrates that low PON1 activity is associated with increased risk of cardiovascular disease [13]. HDL-PON1 antioxidant activity has been correlated with carotid intima-media thickness [14]. The hydrolytic lactonase, arylesterase, and paraoxonase activities of PON1 are all inactivated under oxidative stress [15]. Immunohistochemical analysis has shown accumulation of PON1 in the human lesion as it progresses from fatty streak to advanced lesion [5, 16].

PON2 is expressed in most tissues, including macrophages [5]. Like PON1, PON2 has also been shown to protect vascular cells from oxidative stress, decrease triglyceride accumulation in macrophages [10, 17, 18], play a role against inflammation, and exhibit high acylhomoserine lactone hydrolysis. PON3, like PON1, is associated with HDL but does not exhibit paraoxonase activity and is 200 times less abundant [19].

We recently reported that lipids derived from carotid atherosclerotic plaque (lesion lipids extract—LLE) can accelerate macrophage and lipoprotein oxidation and possess atherogenic properties [20], with the formation of macrophages with foam cell-like appearance [21]. Incubation of recombinant PON1 (rePON1) with LLE reduces lipid carotid plaque atherogenicity [20, 21] but at the same time, linoleic acid hydroperoxide (LA-13OOH) present in the LLE inhibits rePON1 paraoxonase and lactonase activities via reaction of LA-13OOH with the enzyme's cysteine at position 284 (Cys284) [22]. These dual effects between plaque constituents and the elements that are in contact with them, such as blood components (lipids, proteins) circulating via the lesion, led us to further investigate (a) if PONs present in the human carotid plaque [16, 23] are still active and (b) if differences between symptomatic and asymptomatic patients occur within the plaque constituents and between plaque and blood elements.

## 2. Materials and Methods

### 2.1. Materials

N,O-bis(trimethylsilyl) acetamide (BSA), cholesterol, 2-hydroxyquinoline (2HQ), diethyl p-nitrophenyl phosphate (paraoxon), and 4-nitrophenyl acetate were purchased from Sigma-Aldrich. 5-(Thiobutyl)butyrolactone (TBBL) was synthesized in our laboratory by a previously described method [24]. 7*α*-Hydroxycholesterol, 7*β*-hydroxycholesterol, *β*-epoxycholesterol, *α*-epoxycholesterol, 26-hydroxycholesterol, and 7-ketocholesterol were purchased from Steraloids Inc. (Wilton, NH). Recombinant PON1 from *Escherichia coli* was purchased from the Structural Proteomics Center, Weizmann Institute of Science (Rehovot, Israel).

### 2.2. Carotid Plaques

Human carotid plaques were taken from patients undergoing routine endarterectomy in the Department of Vascular Surgery in Carmel Hospital (Haifa, Israel). Both symptomatic and asymptomatic patients underwent surgery under local anesthesia. Patients were considered symptomatic if they had experienced stroke, transient ischemic attack, or amaurosis fugax ipsilateral to the carotid lesion being studied. Complete atherosclerotic plaques were removed, including the common internal and external carotid sections and were immediately placed in saline and kept at −80°C. All plaques were approved for research by the Helsinki Committee regulations, with patient consent (Helsinki approval number 3071). Lesion samples were laid on filter paper to absorb the liquid and then weighed and ground to a powder under liquid nitrogen. The powder was extracted at 4°C for 30 min with PON activity buffer (1 mM CaCl_2_ in 50 mM Tris-HCl, pH 8.0) with 0.1% (v/v) Protease Inhibitor Cocktail and 1 mM PMSF (1 mL for 400 mg tissue), and centrifuged at 10,000 g for 10 min at 4°C. The supernatant was removed and used for determination of protein levels by DC protein assay (Bio-Rad) and for PON activities. Precipitate was ground again under liquid nitrogen and extracted with ethyl acetate. Ethyl acetate was evaporated, and the LLE was dissolved in DMSO to a final concentration of 50 mg/mL and used for detection of lipids and oxidized lipids by liquid chromatography-mass spectrometry (LC-MS) or gas chromatography (GC-MS).

### 2.3. Recombinant PON1 (rePON1)

rePON1 was generated in *E. coli* by directed evolution as described previously [25]. PON1 storage buffer (50 mM Tris, pH 8.0, 50 mM NaCl, 1 mM CaCl_2_, and 0.1% v/v tergitol) was supplemented with 0.02% (w/v) sodium azide and stored at 4°C.

### 2.4. Lactonase Activity

Protein (40 *μ*g) from the plaque homogenate, 0.1 *μ*g/mL rePON1, or 1 : 20 diluted serum was taken for a total reaction volume of 200 *μ*L. Lactonase activity was measured using TBBL as the substrate [24]. Initial rates of hydrolysis were determined spectrophotometrically at 405 nm. The assay mixture included 1 mM TBBL and 1 mM CaCl_2_ in 50 mM Tris-HCl, pH 8.0. Nonenzymatic hydrolysis of TBBL was subtracted from the total rate of hydrolysis. One unit of lactonase activity was equal to 1 *μ*mol of TBBL hydrolyzed/min mL.

### 2.5. Paraoxonase Activity

Protein (100 *μ*g) from the plaque homogenate or 0.2 *μ*g/mL rePON1 was taken for a total reaction volume of 200 *μ*L. Paraoxonase activity was measured using paraoxon as the substrate. Initial rates of hydrolysis were determined spectrophotometrically at 405 nm. The basal assay mixture included 2 mM paraoxon and 1 mM CaCl_2_ in 50 mM glycine/NaOH buffer, pH 10.5. Nonenzymatic hydrolysis of paraoxon was subtracted from the total rate of hydrolysis. One unit of PON1 paraoxonase activity was equal to 1 nmol of paraoxon hydrolyzed/min mL [26].

### 2.6. Arylesterase Activity

Protein (60 *μ*g) from the plaque homogenate or 0.1 *μ*g/mL rePON1 was taken for a total reaction volume of 200 *μ*L. Arylesterase activity was measured using 4-nitrophenyl acetate as the substrate. Initial rates of hydrolysis were determined spectrophotometrically at 405 nm. The assay mixture included 3 mM 4-nitrophenyl acetate and 1 mM CaCl_2_ in 50 mM Tris-HCl, pH 8.0. Nonenzymatic hydrolysis of 4-nitrophenyl acetate was subtracted from the total rate of hydrolysis. One unit of arylesterase activity was equal to 1 *μ*mol of 4-nitrophenyl acetate hydrolyzed/min mL.

### 2.7. Plaque Triglyceride Mass

Plaque triglyceride mass was determined using the Serum Triglyceride Kit from Sigma (Rehovot, Israel). In short, plaque lipids were extracted in ethyl acetate and dried under a nitrogen stream. Dry samples were dissolved in DMSO (50 mg/mL), and 50 *μ*L was added to 1 mL of glycerol reagent and 4 mL triglyceride reagent, vortexed and incubated for 10 min at 37°C. Triglyceride levels were determined at 540 nm according to a glycerol standard.

### 2.8. Oxysterol Detection by GC-MS

Samples were first subjected to hydrolysis to convert all sterol esters into their free form. The dry residue of the extracted sample was dissolved in 0.5 mL KOH solution (20% KOH in a mixture of MeOH : DDW 70 : 30) and mixed for 3 h at 21°C. Two volumes of diethyl ether were added and the pH was adjusted to 5 with 0.5 mL of citric acid (20% in DDW). The upper organic phase was removed and the liquid phase was extracted with another portion of 2 mL diethyl ether. The organic layers were combined, treated with sodium sulfate, and evaporated to dryness under nitrogen purge. Dried extracts were subjected to the silylating reagent BSA dissolved in 1,4-dioxane (dried on 4 Å molecular sieves and passed through aluminum oxide) and heated to 80°C for 60 min. Oxysterol was detected as previously described [3]. Briefly, standards or dried extracts were subjected to 200 *μ*L BSA, followed by the addition of 300 *μ*L 1,4-dioxane (treated as before) and heated to 80°C for 60 min. Samples were detected by GC-MS in a total ion monitoring (TIM) mode, and 2–4 of the most representative ions were selected for reinjection in single ion monitoring (SIM) mode. The mean quantity of each oxysterol was calculated from calibration curves of its standard.

### 2.9. Analyzing FFAs and Oxidized FFAs by LC-MS/MS

FFAs were quantified by LC-MS/HPLC (Waters 2790) connected to an MS (Micromass Quattro UltimaMS, UK). The HPLC column was a 3.5 mm C18 ODS XTerra column (Waters). MS analysis of the FFAs was performed in SIM mode, using electron spray negative ions. MS/MS analysis of the oxidized products was performed in scan and daughter modes, using ES^−^. The source temperature of the MS was set at 120°C, with a cone gas flow of 22 L/h and a desolvation gas flow of 400 L/h. Peak spectra were monitored between *m/z* 50 and 350. Collision-induced dissociation MS was performed, with a collision energy of 20 eV and 3–3.5 kV capillary voltage. Multiple-reaction monitoring was performed under the same conditions used to quantify the oxidized products.

### 2.10. Statistical Analysis

Statistical analysis was performed using the Student paired or heteroscedastic *t*-test when comparing the means of two groups. Linear regression was calculated using GraphPad Prism 4 software. Each experiment was repeated, separately, at least three times (*n* ≥ 3). Results are presented as mean ± SEM. Clinical parameters were analyzed using GraphPad Prism 4 software by Contingency table with chi-square test.

## 3. Results

### 3.1. Plaque PON Activity

**Figure 1:**
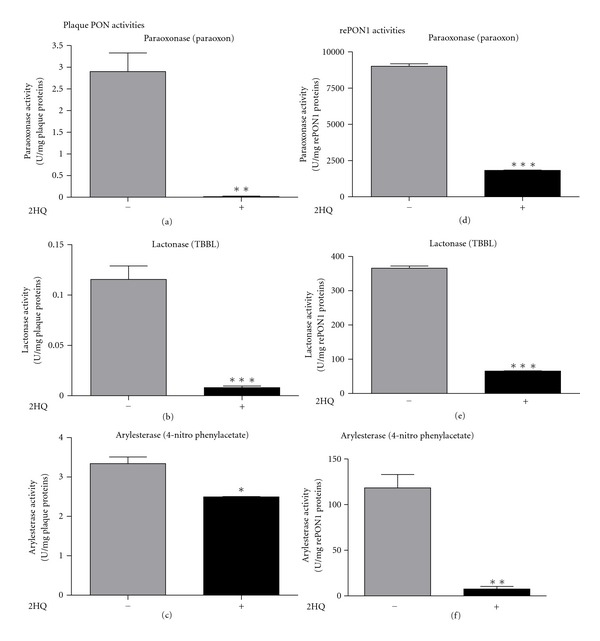
Hydrolytic activities of paraoxonases (PONs) in human carotid plaque homogenate and of recombinant PON1 (rePON1). (a): Homogenate paraoxonase (paraoxon), (b): lactonase (TBBL), and (c): arylesterase (4-nitrophenyl acetate) activities; (d): rePON1 paraoxonase, (e): lactonase, and (f): arylesterase activities, with or without 2-hydroxyquinoline (2HQ).

Immunohistochemical analysis has recently revealed the presence of PON1, PON3 [16], and PON2 [23] in carotid lesions. However, such analyses do not indicate whether the enzymes in the plaque still possess hydrolytic activity. As part of our ongoing research into the chemical composition of the human carotid plaque, the mechanism by which plaque components interrelate, and their possible dual effects with blood components, we tested whether plaque PONs are still active. Human carotid lesions were ground to a powder under liquid nitrogen. The powder was extracted with Tris buffer and then centrifuged (see Section 2). The supernatant was used for determination of PON paraoxonase, lactonase and arylesterase activities, with or without the addition of the PON1 inhibitor 2HQ. All three PON activities were preserved in the plaque homogenate, and 2HQ almost completely inhibited paraoxonase and lactonase activities (from 2.9 to 0.014 and from 0.115 to 0.008 U/mg protein, resp., Figures 1(a) and 1(b)), whereas it only slightly decreased arylesterase activity (from 3.3 to 2.5 U/mg protein) (Figure 1(c)). The effects of 2HQ on PON's hydrolytic properties were reexamined with rePON1. 2HQ inhibited rePON1 paraoxonase, lactonase, and arylesterase activities almost completely (from  9006 ± 161  to 1819 ± 30,  365 ± 6  to 65.6 ± 0.7, and  118 ± 14  to 9.9 ± 2.8 U/mg rePON1 protein, resp.) (Figures 1(d)–1(f)). These results indicated that PONs that are present in the plaques preserve all three of their hydrolytic activities.

### 3.2. Symptomatic versus Asymptomatic Patients

Atherosclerotic patients have been shown to have less PON1 activity in their blood than healthy subjects [27]. Since symptomatic patients have some features of vulnerable plaques, their plaques are considered to be more severe than those of asymptomatic patients [7, 28, 29]. In the present study, clinical parameters were compared between patients with hypertension, hyperlipidemia, or being treated with statins or antihypertensive drugs: no significant differences were found between symptomatic and asymptomatic patients in these criteria. The symptomatic group included more diabetic subjects than the asymptomatic group (Table 1). Differences in plaque PON activity between symptomatic and asymptomatic patients were assessed. Surprisingly, there was no significant difference in plaque lactonase activity between the two groups (Table 2). Serum PON1 activity, as expected, was lower (15%) in the symptomatic patients than in the asymptomatic patients, but the difference was not significant (*P* = 0.14) (Table 2). Atherogenic components were then compared between symptomatic and asymptomatic patients, including levels of triglyceride, oxysterol, and LA-13OOH in the plaque lipid extract, and levels of lipoproteins and hemoglobin A1c (HbA1c) in individual patients' blood. The levels of various lipids and oxidized lipids in the plaque extracts were determined by MS. No significant differences were found in the amounts of detected oxysterols (5,6-*α*- and *β*-epoxy cholesterol, 7*α*-OH and 7*β*-OH cholesterol, 7-keto cholesterol, and 26-OH cholesterol) or triglycerides. In contrast, symptomatic patients had significantly higher (135%) amounts of LA-13OOH in their plaques than asymptomatic patients (Table 2). In addition, while there was no significant difference in the amounts of total cholesterol, LDL cholesterol, or triglyceride in the blood, symptomatic patients had significantly less (28%) HDL cholesterol than the asymptomatic group (Table 2). Symptomatic patients also had more (18%) HbA1c in their blood than asymptomatic patients, although this difference was only marginally significant (Table 2).

### 3.3. Correlations between Plaque LA-13OOH and Elements in the Blood of the Same Individual

**Figure 2:**
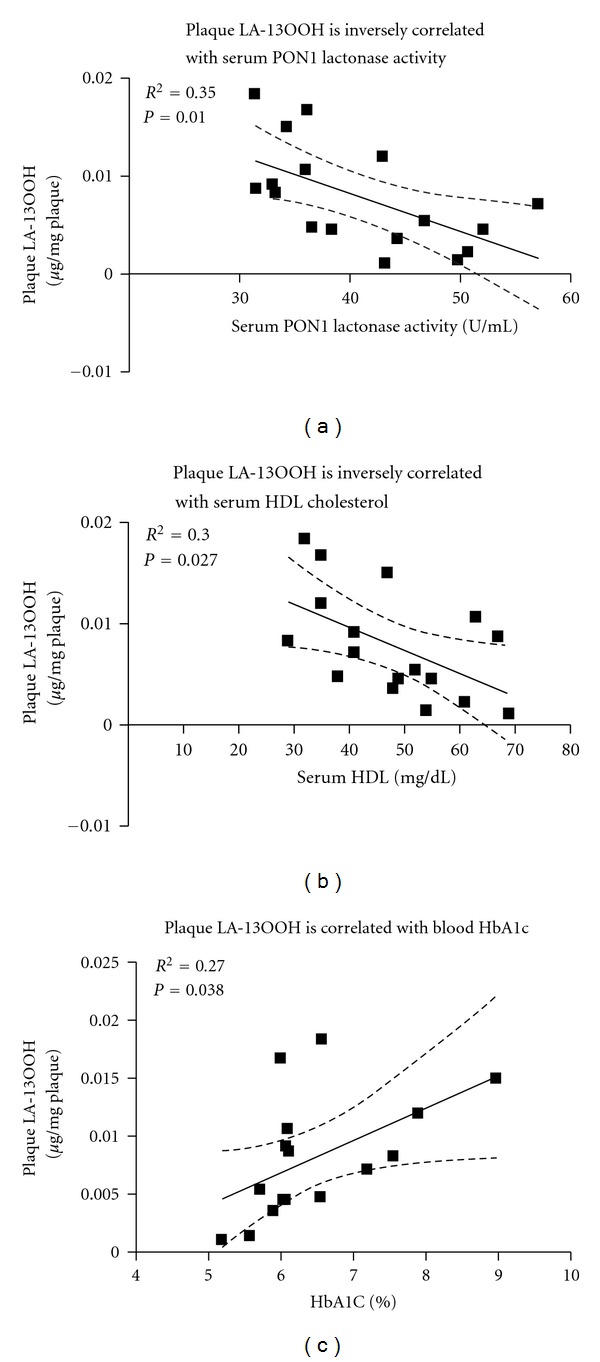
Human plaque linoleic acid hydroperoxide (LA-13OOH) level versus serum HDL, serum PON1 activity and blood hemoglobin (Hb) A1c. Human plaque LA-13OOH is inversely correlated with (a): serum PON1 lactonase activity and (b): serum HDL, but (c): positively correlated with blood HbA1c.

Previous studies from our group have shown that LA-13OOH (with the hydroperoxide at position 13 of linoleic acid) inhibits PON1 by specific interaction with the enzyme's Cys284. Thus, LA-13OOH is considered an atherogenic factor in the plaque which can augment oxidative stress and progression of atherosclerosis [22]. This led us to correlate LA-13OOH level in the plaque with other atherogenic and antiatherogenic elements in the blood of the same patient, to assess the possible correlation between plaque status and a specific component in the blood. Results showed that plaque LA-13OOH level is indeed inversely correlated with two antiatherogenic elements in the blood: serum PON1 lactonase activity (*R* ^2^ = 0.35, *P* = 0.01, Figure 2(a)) and HDL cholesterol (*R* ^2^ = 0.3, *P* = 0.027, Figure 2(b)). In addition, a direct correlation was found between the two atherogenic elements, LA-13OOH and HbA1c (*R* ^2^ = 0.27, *P* = 0.038, Figure 2(c)).

## 4. Discussion

Atherogenesis is accompanied by the accumulation of oxidized lipids in the arterial wall. The antioxidant enzyme PON1 lowers lipid peroxide levels [30], protects arterial cell walls (as well as endothelial cells, smooth muscle cells, and macrophages) and lipoproteins (LDL and HDL) from oxidation, inhibits oxidized LDL uptake by macrophages [9, 10], and shows anti-inflammatory activities [31, 32]. Thus, the presence of PON1, PON2, and PON3 in the human plaque may have an important role in decreasing atherosclerotic progression. Immunohistochemical analysis of human plaque has revealed the presence of PONs in the lesion; during lesion progression, there is a shift of PON1 and PON3 from smooth muscle cells to macrophages [16], whereas arterial PON2 level decreases [23]. Hence, it is important to assess whether the PONs identified immunohistochemically in the plaque are still active. Our current results show that the PONs are indeed still active in the plaque homogenate, preserving their catalytic paraoxonase, arylesterase, and lactonase activities. Among these activities, only PON1 can hydrolyze paraoxon, hence we concluded that human carotid lesion has active PON1. PON1 may thus act as a potent reducing antioxidant enzyme not only in the serum, but also within the plaque, leading to attenuated atherosclerotic progression. In addition, while 2HQ inhibited rePON1 arylesterase almost completely but only slightly decreased arylesterase in the homogenate, we can conclude that in the latter, other esterases are present and PON's contribution is only minor.

**Figure 3:**
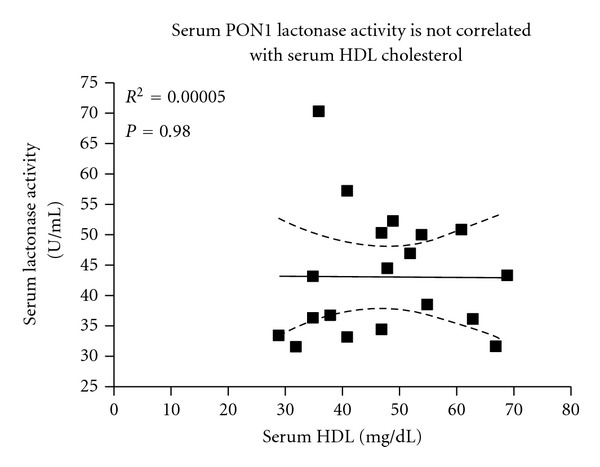
Serum PON1 activity versus serum HDL cholesterol. Serum PON1 activity is not correlated with serum HDL cholesterol levels.

A distinction between symptomatic and asymptomatic patients through the identification of biomarkers could provide information on symptom occurrence. Such biomarkers are not yet available but are needed to make appropriate decisions on the type of intervention required [8]. Previous studies from our group have shown that LA-13OOH is present in the lipid extract of the human carotid plaque, and that it specifically inhibits rePON1 activity in a dose- and time-dependent manner. During PON1's interaction with lesion LA-13OOH, the enzyme displayed a peroxidase-type of catalysis, reducing LA-13OOH to LA-OH (hydroxide) via the PON1 amino acid Cys284 [22]. Thus, the levels of LA-13OOH in symptomatic and asymptomatic patients were compared and correlated with antiatherogenic compounds in the plaque, or in the serum derived from these patients. Symptomatic patients had significantly higher levels of LA-13OOH in their plaques than their asymptomatic counterparts. In addition, LA-13OOH levels in the plaque were significantly inversely correlated with serum PON1 activity (Figure 2(a)) and with serum HDL cholesterol (Figure 2(b)). Although PON1 is an HDL-associated enzyme, HDL particles are highly heterogeneous in their structure, intravascular metabolism, and biological activities [33]. Furthermore, PON1 is only present on a relatively small fraction of the HDL particles, mostly on the HDL3 subfraction. Thus, the amount of the HDL in the blood is not necessarily correlated with serum PON1 activity. Indeed, in this study, serum PON1 lactonase activity was not correlated with serum HDL levels (Figure 3). These results indicate that LA-13OOH might be affected, independently, by both serum PON1 and serum HDL level. As plaque LA-13OOH has been shown to interact with and inhibit rePON1 [22], and since PON1 is present and active, in both blood and the atherosclerotic plaque, we determined PON1 activity in serum and plaques derived from the same individuals and compared them in symptomatic versus asymptomatic patients. Our results showed no differences in plaque PON activity between symptomatic and asymptomatic atherosclerotic patients (*P* = 0.8). Serum PON1 activity in the symptomatic patients was lower than in the asymptomatic patients, although this difference was not significant (*P* = 0.14) (Table 2). Plaque PON activity may be attributed to both PON1 and the intracellular enzyme PON2, which may not be affected by the presence of LA-13OOH. In addition, it has been previously shown that inhibition of rePON1 by LA-13OOH can be prevented if certain thiols, such as the amino acid cysteine, are present, and that PON1 inhibition by LA-13OOH can be partially recovered if a thiol is added to the incubation system. LA-13OOH probably oxidizes the PON1 Cys284 to sulfenic acid, which can then be further oxidized to sulfinic and sulfonic acid derivatives. In the presence of thiol, the sulfenic acid derivative of Cys284 can be reduced back to thiol; however, if the oxidation proceeds further, addition of an external thiol can no longer reverse the reaction [22]. We can, therefore, hypothesize that the presence of free cysteine in the blood and in the plaque may also prevent LA-13OOH-induced PON1 inactivation.

 In accordance with Carr et al. [29], we did not observe significant differences in serum cholesterol or triglyceride levels between symptomatic and asymptomatic patients, or in triglyceride or oxysterol levels in the plaques themselves (Table 2).

In addition to their higher levels of plaque LA-13OOH and lower levels of serum HDL relative to asymptomatic patients, there are more diabetics among the symptomatic patients (Table 1), as characterized by a higher concentration of blood HbA1c (Table 2). HbA1c is a glycated hemoglobin, which is highly prone to oxidation and its level is linked to oxidative stress. Diabetic patients are a heterogeneous population that may differ in their exposure to risk factors. For example, haptoglobin (Hp) genotype is an independent risk factor for vascular complications in diabetes [34]. This study suggests LA-13OOH as a possible risk factor for diabetic atherosclerosis development and complications, as analyses of plaque LA-13OOH and blood HbA1c revealed a direct correlation between these two atherogenic elements. Levels of HbA1c are strongly correlated with mean blood glucose [35], and high glucose concentration is known to increase oxidative stress [36]. HbA1c is also correlated with lipid peroxidation values, as shown in type 1 diabetic patients [37], as well as with plasma aldehydes (malondialdehydes) in type 2 diabetics [38, 39]. Furthermore, Hussein et al. [40] recently showed that the lag time required for the initiation of LDL oxidation (in the presence of copper ions) is inversely correlated with HbA1c concentration (mainly when HbA1c < 7.3%). Moreover, incubation of red blood cell hemolysate with increasing concentrations of glucose and with LDL or oxidized LDL results in an increased concentration of HbA1c. Thus, the increased tendency for LDL to undergo lipid peroxidation in diabetic patients contributes to increased levels of blood HbA1c. This further emphasizes the strong association between HbA1c and oxidative stress, which is in agreement with the present findings pointing to a direct link between the atherogenic compounds in the atherosclerotic lesions and serum, such as LA-13OOH and blood HbA1c. In addition, it is in line with the inverse relationships shown for serum HDL and PON1 activity, which are both antiatherogenic elements of the blood.

 In conclusion, LA-13OOH levels in plaques from symptomatic patients are higher than those in asymptomatic patients' plaques. The high levels of plaque LA-13OOH are correlated with low levels of serum HDL, low levels of serum PON1 lactonase activity, and increased concentrations of blood HbA1c, all of which further accelerate atherosclerotic progression. Thus, the levels of PON1 activities, HDL concentration, and HbA1c content could serve as suitable biomarkers to assess LA-13OOH level in human carotid plaques.

## Figures and Tables

**Table 1:** Symptomatic and asymptomatic clinical parameters.

	Symptomatic	Asymptomatic	*P* value
Age (y)	68 ± 1.6	72 ± 2.5	0.2
Hypertension	5/6 (83%)	10/13 (77%)	0.75
Hyperlipidemia	4/6 (67%)	10/13 (77%)	0.64
Treated with statins	5/6 (83%)	11/13 (85%)	0.94
Treated with antihypertensive drugs	4/6 (67%)	9/13 (69%)	0.91
Diabetes	3/6 (50%)	1/13 (8%)	0.035

**Table 2:** Atherogenic and antiatherogenic elements in the plaques and blood of symptomatic and asymptomatic patients.

		Symptomatic (*n* = 6)	Asymptomatic (*n* = 13)	* P *value
Plaque	PON lactonase activity (U/mg proteins)	0.16 ± 0.035 (*n* = 8)	0.18 ± 0.054 (*n* = 9)	N.S.
	Triglyceride (% of LLE)	3.4 ± 1.07	2.37 ± 0.37	N.S.
	7-keto (% of cholesterol)	0.38 ± 0.19	0.25 ± 0.05	N.S.
	26-OH (% of cholesterol)	1.16 ± 0.27	1.29 ± 0.19	N.S.
	7*α*-OH (% of cholesterol)	0.087 ± 0.015	0.085 ± 0.01	N.S.
	7*β*-OH (% of cholesterol)	0.075 ± 0.01	0.08 ± 0.009	N.S.
	*β*-epoxy (% of cholesterol)	0.096 ± 0.01	0.114 ± 0.02	N.S.
	*α*-epoxy (% of cholesterol)	0.055 ± 0.004	0.069 ± 0.008	N.S.
	LA-13OOH (*μ*g/mg plaque)	0.012 ± 0.002	0.0053 ± 0.0009	0.019

Serum	PON1 lactonase activity (U/mL)	38 ± 3.2	45 ± 2.9	N.S.
	HbA1c (%)	7.17 ± 0.48	6.04 ± 0.15	0.065
	HDL (mg/dL)	37.8 ± 3.35	51.7 ± 3.06	0.009
	LDL (mg/dL)	79 ± 13.2	90.9 ± 4.4	N.S.
	Cholesterol (mg/dL)	158.6 ± 11.2	168.08 ± 6.4	N.S.
	Triglyceride (mg/dL)	164.8 ± 27	127.2 ± 15.2	N.S.

LLE: lesion lipid extract. Results are presented as mean ± SEM.

## References

[1] Badimon JJ, Fuster V, Chesebro JH, Badimon L (1993). Coronary atherosclerosis: a multifactorial disease. *Circulation*.

[2] Witztum JL, Steinberg D (1991). Role of oxidized low density lipoprotein in atherogenesis. *Journal of Clinical Investigation*.

[3] Vaya J, Aviram M, Mahmood S (2001). Selective distribution of oxysterols in atherosclerotic lesions and human plasma lipoproteins. *Free Radical Research*.

[4] Carpenter KL, Taylor SE, Van der Veen C, Williamson BK, Ballantine JA, Mitchinson MJ (1995). Lipids and oxidised lipids in human atherosclerotic lesions at different stages of development. *Biochimica et Biophysica Acta*.

[5] Judit Marsillach BM, Fransec MM, Beltran R, Joven J, Camp J Immunohistochemical analysis of Paraxoxnase-1,2 and 3 in human atheroma plaques.

[6] Mackness B, Hunt R, Durrington PN, Mackness MI (1997). Increased immunolocalization of paraoxonase, clusterin, and apolipoprotein A-I in the human artery wall with the progression of atherosclerosis. *Arteriosclerosis, Thrombosis, and Vascular Biology*.

[7] Mughal MM, Khan MK, DeMarco JK (2011). Symptomatic and asymptomatic carotid artery plaque. *Expert Reviews*.

[8] Hermus L, Lefrandt JD, Tio RA, Breek JC, Zeebregts CJ (2010). Carotid plaque formation and serum biomarkers. *Atherosclerosis*.

[9] Rosenblat M, Volkova N, Aviram M (2010). Pomegranate juice (PJ) consumption antioxidative properties on mouse macrophages, but not PJ beneficial effects on macrophage cholesterol and triglyceride metabolism, are mediated via PJ-induced stimulation of macrophage PON2. *Atherosclerosis*.

[10] Aviram M, Rosenblat M (2004). Paraoxonases 1, 2, and 3, oxidative stress, and macrophage foam cell formation during atherosclerosis development. *Free Radical Biology and Medicine*.

[11] Shih DM, Welch C, Lusis AJ (1995). New insights into atherosclerosis from studies with mouse models. *Molecular Medicine Today*.

[12] Mackness B, Quarck R, Verreth W, Mackness M, Holvoet P (2006). Human paraoxonase-1 overexpression inhibits atherosclerosis in a mouse model of metabolic syndrome. *Arteriosclerosis, Thrombosis, and Vascular Biology*.

[13] Jarvik GP, Rozek LS, Brophy VH (2000). Paraoxonase (PON1) phenotype is a better predictor of vascular disease than is *PON*1_192_ or *PON*1_55_ genotype. *Arteriosclerosis, Thrombosis, and Vascular Biology*.

[14] Harangi M, Seres I, Magyar MT (2008). Association between human paraoxonase 1 activity and intima-media thickness in subjects under 55 years of age with carotid artery diseases. *Cerebrovascular Diseases*.

[15] Aviram M, Rosenblat M, Billecke S (1999). Human serum paraoxonase (PON 1) is inactivated by oxidized low density lipoprotein and preserved by antioxidants. *Free Radical Biology and Medicine*.

[16] Marsillach J, Camps J, Beltran-Debón R (2011). Immunohistochemical analysis of paraoxonases-1 and 3 in human atheromatous plaques. *European Journal of Clinical Investigation*.

[17] Rosenblat M, Coleman R, Reddy ST, Aviram M (2009). Paraoxonase 2 attenuates macrophage triglyceride accumulation via inhibition of diacylglycerol acyltransferase 1. *Journal of Lipid Research*.

[18] Horke S, Witte I, Wilgenbus P, Krüger M, Strand D, Förstermann U (2007). Paraoxonase-2 reduces oxidative stress in vascular cells and decreases endoplasmic reticulum stress-induced caspase activation. *Circulation*.

[19] Draganov DI, Stetson PL, Watson CE, Billecke SS, La Du BN (2000). Rabbit serum paraoxonase 3 (PON3) is a high density lipoprotein-associated lactonase and protects low density lipoprotein against oxidation. *Journal of Biological Chemistry*.

[20] Tavori H, Aviram M, Khatib S (2009). Human carotid atherosclerotic plaque increases oxidative state of macrophages and low-density lipoproteins, whereas paraoxonase 1 (PON1) decreases such atherogenic effects. *Free Radical Biology and Medicine*.

[21] Tavori H, Aviram M, Khatib S (2011). Paraoxonase 1 protects macrophages from atherogenicity of a specific triglyceride isolated from human carotid lesion. *Free Radical Biology and Medicine*.

[22] Tavori H, Aviram M, Khatib S (2011). Human carotid lesion linoleic acid hydroperoxide inhibits paraoxonase 1 (PON1) activity via reaction with PON1 free sulfhydryl cysteine 284. *Free Radical Biology and Medicine*.

[23] Fortunato G, Di Taranto MD, Bracale UM (2008). Decreased paraoxonase-2 expression in human carotids during the progression of atherosclerosis. *Arteriosclerosis, Thrombosis, and Vascular Biology*.

[24] Khersonsky O, Tawfik DS (2006). Chromogenic and fluorogenic assays for the lactonase activity of serum paraoxonases. *ChemBioChem*.

[25] Harel M, Aharoni A, Gaidukov L (2004). Structure and evolution of the serum paraoxonase family of detoxifying and anti-atherosclerotic enzymes. *Nature Structural and Molecular Biology*.

[26] Gaidukov L, Tawfik DS (2005). High affinity, stability, and lactonase activity of serum paraoxonase PON1 anchored on HDL with ApoA-I. *Biochemistry*.

[27] Kotur-Stevuljevic J, Spasic S, Stefanovic A (2006). Paraoxonase-1 (PON1) activity, but not PON1Q192R phenotype, is a predictor of coronary artery disease in a middle-aged Serbian population. *Clinical Chemistry and Laboratory Medicine*.

[28] Golledge J, Greenhalgh RM, Davies AH (2000). The symptomatic carotid plaque. *Stroke*.

[29] Carr S, Farb A, Pearce WH (1996). Atherosclerotic plaque rupture in symptomatic carotid artery stenosis. *Journal of Vascular Surgery*.

[30] Aviram M, Hardak E, Vaya J (2000). Human serum paraoxonases (PON1) Q and R selectively decrease lipid peroxides in human coronary and carotid atherosclerotic lesions: PON1 esterase and peroxidase-like activities. *Circulation*.

[31] Mackness MI, Arrol S, Abbott C, Durrington PN (1993). Protection of low-density lipoprotein against oxidative modification by high-density lipoprotein associated paraoxonase. *Atherosclerosis*.

[32] Watson AD, Berliner JA, Hama SY (1995). Protective effect of high density lipoprotein associated paraoxonase. Inhibition of the biological activity of minimally oxidized low density lipoprotein. *Journal of Clinical Investigation*.

[33] Camont L, Chapman MJ, Kontush A (2011). Biological activities of HDL subpopulations and their relevance to cardiovascular disease. *Trends in Molecular Medicine*.

[34] Asleh R, Levy AP (2005). In vivo and in vitro studies establishing haptoglobin as a major susceptibility gene for diabetic vascular disease.. *Vascular Health and Risk Management*.

[35] (1987). Diabetes control and complications trial (DCCT): results of feasibility study. The DCCT research group. *Diabetes Care*.

[36] Bierman EL (1992). George lyman duff memorial lecture. Atherogenesis in diabetes. *Arteriosclerosis, Thrombosis*.

[37] Ruiz C, Alegría A, Barberá R, Farré R, Lagarda MJ (1999). Lipid peroxidation and antioxidant enzyme activities in patients with type 1 diabetes mellitus. *Scandinavian Journal of Clinical and Laboratory Investigation*.

[38] Altomare E, Vendemiale G, Chicco D, Procacci V, Cirelli F (1992). Increased lipid peroxidation in Type 2 poorly controlled diabetic patients. *Diabete et Metabolisme*.

[39] Velazquez E, Winocour PH, Kesteven P, Alberti KGMM, Laker MF (1991). Relation of lipid peroxides to macrovascular disease in Type 2 diabetes. *Diabetic Medicine*.

[40] Hussein OA, Gefen Y, Zidan JM (2007). LDL oxidation is associated with increased blood hemoglobin A1c levels in diabetic patients. *Clinica Chimica Acta*.

